# Pyrimidine-5-carbonitrile based potential anticancer agents as apoptosis inducers through PI3K/AKT axis inhibition in leukaemia K562

**DOI:** 10.1080/14756366.2022.2051022

**Published:** 2022-03-29

**Authors:** Nehad M. El-Dydamony, Rana M. Abdelnaby, Rasha Abdelhady, Omaima Ali, Mohamed I. Fahmy, Rasha R. Fakhr Eldeen, Amira A. Helwa

**Affiliations:** a Pharmaceutical Chemistry Department, College of Pharmaceutical Sciences and Drug Manufacturing, Misr University for Science and Technology, 6th of October City, Egypt; b Pharmaceutical Chemistry Department, Faculty of Pharmacy, Heliopolis University, Cairo, Egypt; c Pharmacology and Toxicology Department, Faculty of Pharmacy, Fayoum University, Fayoum, Egypt; d Cell Line Unit, Egyptian Drug Authority (EDA), Cairo, Egypt; e Pharmacology and Toxicology Department, Faculty of Pharmacy, Heliopolis University, Cairo, Egypt; f Biochemistry Department, College of Pharmaceutical Sciences and Drug Manufacturing, Misr University for Science and Technology, 6th of October City, Egypt; g Pharmaceutical Organic Chemistry Department, College of Pharmaceutical Sciences and Drug Manufacturing, Misr University for Science and Technology, 6th of October City, Egypt

**Keywords:** Pyrimidine-5-carbonitrile, leukaemia (K562), breast cancer (MCF-7), PI3K/AKT pathway, apoptosis

## Abstract

A novel series of 4-(4-Methoxyphenyl)-2-(methylthio)pyrimidine-5-carbonitrile was developed linked to an aromatic moiety *via N*-containing bridge and then evaluated for their cytotoxic activity against MCF-7 and K562 cell lines. Seven compounds exhibited the highest activity against both cell lines where compounds **4d** and **7f** were the most active against K562 cell line. Exploring their molecular mechanisms by enzyme inhibition assay on PI3Kδ/γ and AKT-1 showed that compound **7f** was promising more than **4d** with IC_50_ = 6.99 ± 0.36, 4.01 ± 0.55, and 3.36 ± 0.17 uM, respectively. Also, flowcytometric analysis revealed that **7f** caused cell cycle arrest at S-phase followed by caspase 3 dependent apoptosis induction. Mechanistically, compound **7f** proved to modulate the expression of PI3K, p-PI3K, AKT, p-AKT, Cyclin D1, and NFΚβ. Furthermore, *in-vivo* toxicity study indicated good safety profile for **7f**. These findings suggest that the trimethoxy derivative **7f** has strong potential as a multi-acting inhibitor on PI3K/AKT axis targeting breast cancer and leukaemia.

## 1. Introduction

Cancer is a devastating group of diseases that WHO announced it as the first or second leading cause of death worldwide in ages before 70 years. Consequently, the global burden of cancer incidence and mortality rates have unprecedented surpass according to the GLOBOCAN 2020 report. It was estimated that there were 19.3 million new cases and almost 10.0 million cancer deaths occurred in 2020 and expected to be 28.4 million cases in 2040 with a 47% rise from 2020. Thus, an effective control for cancer dissemination is an imminent need either through prevention or therapeutic intervention^1^^,^^2^.

The crucial role of the PI3K/AKT/mTOR (PAM) pathway in cell survival, proliferation, growth, apoptosis, and glycogen metabolism and its frequent activation or over expression in many types of human cancers, such as breast, ovarian, prostatic, lung, gastric, pancreatic cancers, and B-cell lymphomas has put it in the line of promising druggable targets in the war against cancer^3–5^. Moreover, mis-regulation or genomic alterations in PAM pathway develop a secondary resistance that menace most targeted anticancer therapies^6^^,^^7^. Hence, attention has been devoted to developing a new cancer treatment with added benefits of overcoming chemoresistance through targeting this oncogenic pathway.

**Figure 1.**
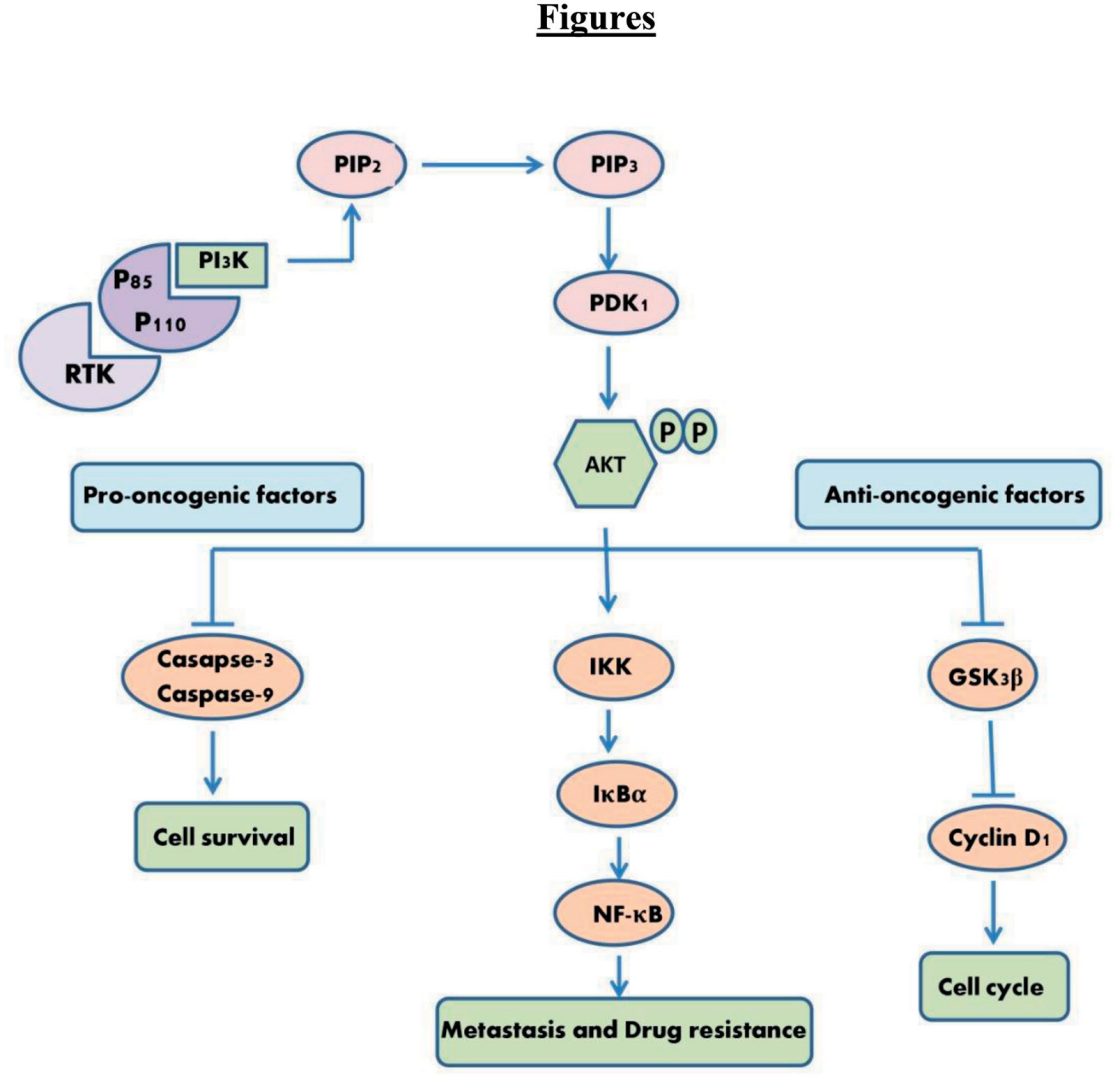
Schematic diagram of PI3K/AKT signalling cascade illustrating some downstream biomarkers under investigation. RTK: receptor tyrosine kinases. Arrows denote activation and bars for inhibition.

Phosphoinositide 3-kinases (PI3Ks) are a family of lipid kinases consisting of three major classes that phosphorylate inositol phospholipids generating the second messenger phosphatidylinositol-3,4,5-triphosphate (PIP3). Of interest is Class I which is frequently associated with cancer development containing four catalytic subunits p110*α* and p110*β* which are expressed in all tissues, p110*γ*, and p110*δ* which were reported to play an important role in haematological malignancies. The formed PIP3 activates the serine/threonine kinase AKT (PKB) which is considered a central node in this pathway allowing phosphorylation at Thr308 or Ser473 by phosphoinositide-dependent kinase (PDK1). Phosphorylated AKT (p-AKT) is involved in the deregulation of apoptosis, proliferation, and cell metastasis by modulating the phosphorylation of several downstream protein substrates that regulate cell growth.^8^^,^^9^ One of the important downstream proteins is cyclin D1 known to promote cell proliferation through mediating cell progression from G1-phase to S-phase. AKT is known to stabilise cyclin D1 through inactive phosphorylation of GSK3Κβ. This results in GSK3Κβ losing its kinase activity to phosphorylate Thr286 in cyclin D1 which in turn inhibits its cytoplasmic proteasomal degradation^6^ (Figure 1).

Previous studies reported that blocking AKT enzyme or inhibiting p-AKT expression only could result in compensatory resistance *via* reactivating PI3K or mTOR. Moreover, researchers documented that phosphorylation of AKT is a transient event that lasts for a short duration followed by re-occurring hyperphosphorylation while treatment is being continued with ATP-competitive inhibitors^10–12^. Whilst, targeting mTOR only by recently developed antitumor agents results in AKT activation then cuts-off the pathway feedback inhibition which attenuates the potential antitumor activity^13^. Consequently, a combination therapy or dual-acting agents on PI3K/AKT is a key approach providing broader efficacy and better patient tolerability compared to other candidates targeting only one component of this pathway^10^.

**Figure 2.**
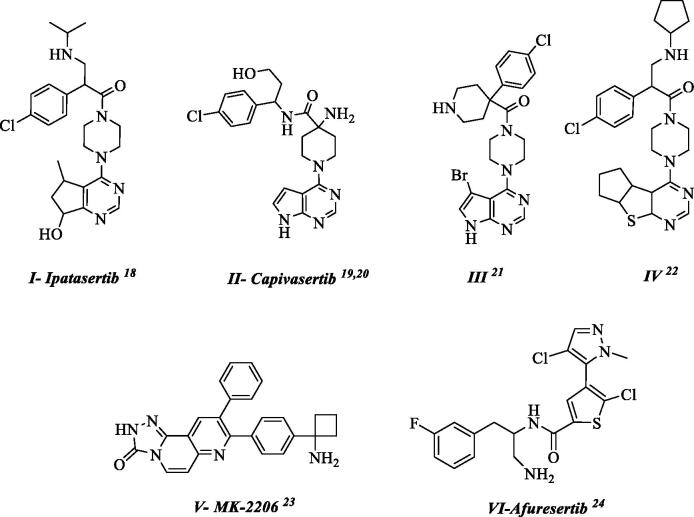
Representative AKT (ATP-competitive and allosteric) inhibitors.

**Figure 3.**
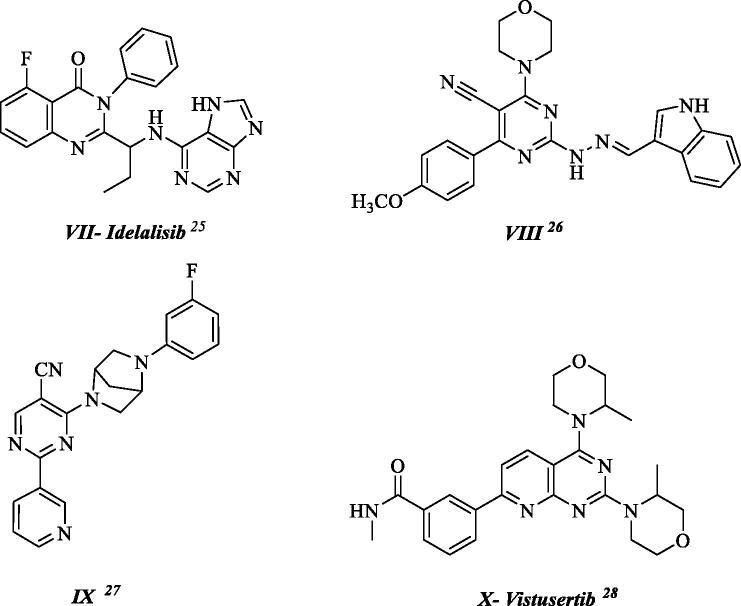
PI3K inhibitors with pyrimidine scaffold.

Out of the huge prevalence of heterocyclic rings in drug development, pyrimidine nucleus has crowned them as it constitutes an integral part in nucleic acid structures. In addition, it establishes a core pharmacophore in natural and synthetic drugs with diverse activity and good safety profile^14^^,^^15^. The antitumor activity of the pyrimidine scaffold is due to its ability to bind to several enzymes, receptors, and target proteins starting from 5-Fluorouracil passing by Imatinib and Methotrexate and even those entered clinical trials nowadays^16–20^ (Figures 2 and 3).

Exemplified inhibitors for this pathway that had been developed and entered clinical trials include: A number of AKT inhibitors acting on two distinct binding sites: ATP-binding site and allosteric pocket of the autoinhibited isoform as shown in Figure 2^18–24^, such as Ipatasertib [GDC0068] **I**^18^ which is a highly selective ATP-competitive pan-Akt inhibitor in phase-III clinical trials to evaluate its effectiveness in triple-negative breast cancer [TNBC] and Capivasertib [AZD5363] **II**^19^^,^^20^ which is also in phase-III trial to further evaluate its efficacy and safety in combination with paclitaxel in first-line treatment of patients with metastatic TNBC. Likewise, the allosteric pan-AKT inhibitor MK-2206 **V**^23^ demonstrated activity in treating patients with relapsed acute myeloid leukaemia (AML) in phase-II clinical studies. Also, Afuresertib **VI**^24^ exhibited a favourable safety profile and clinical activity against haematologic malignancies. Other agents acting on the PAM pathway as presented in Figure 3^25–28^ include: Idelalisib **VII**^25^ a PI3K-δ inhibitor that was approved for the treatment of different types of lymphomas. As well as compound **VIII**^26^ which was developed by our research team having a pyrimidine nucleus known for its diverse anticancer mechanisms substituted with 6-morpholino group as Pan-PI3K inhibitor and apoptosis inducer.

**Figure 4.**
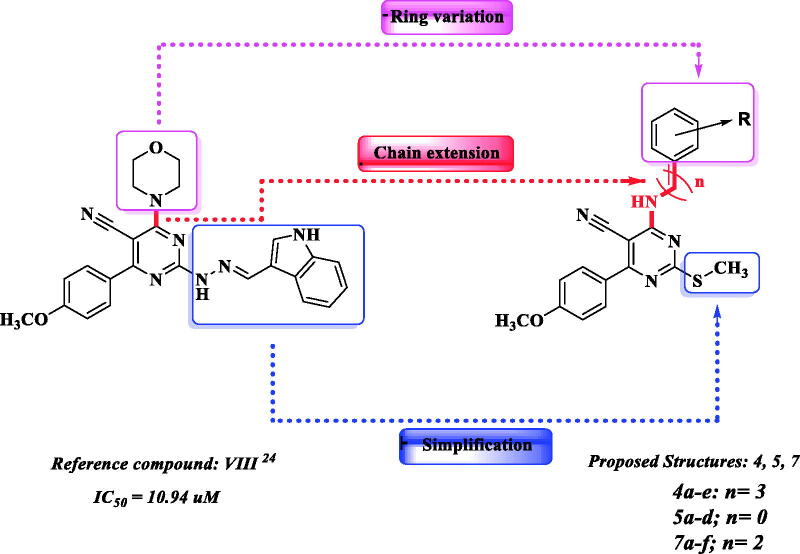
The design strategy of the proposed structures based on the lead compound **VIII**.

In continuing our research work on developing pyrimidine derivatives acting on cell proliferation and apoptosis induction through PI3K/AKT axis inhibition, this work focussed on preparing new analogues derived from our previously reported lead compound (**VIII**)^26^ keeping (4–(4-methoxyphenyl)-2-(methylthio)pyrimidine-5-carbonitrile) as the main core. Moreover, to study the effect of C-4 substitution on enzyme inhibition, ring variation and chain extension strategies were adopted *via* linking the pyrimidine core to substituted aryl moieties through different amino bridges one to four atoms in distance replacing the morpholine ring. Also, replacing indolo-hydrazino moiety at C-2 with small methylthio group to render the molecules more linear to fit with AKT pharmacophoric features^11^^,^^25^ keeping the essential PI3K requirements as illustrated in Figure 4.

Since that pathway has a very obvious implication in breast cancer and leukemia^4^^,^^5^. The *in-vitro* cytotoxic activity of the newly synthesised compounds was assayed against breast cancer (MCF-7) and acute leukaemia (K562) cell lines. To explore the underlying molecular mechanism of these compounds; PI3Kδ, PI3Kγ, and AKT-1 enzyme inhibition assays were done followed by cell cycle analysis, apoptosis induction, and caspase 3 evaluation using ELISA. In addition, western blotting assay for PI3K, p-PI3K, AKT, p-AKT, Cyclin D1, and NFΚβ were carried for the most active compound to see the effect on the downstream proteins of this pathway. Then, molecular simulation studies were performed to clarify the binding behaviour of the most active derivative with the nominated enzyme isoforms and to predict its pharmacokinetics. Finally, *in-vivo* toxicity study was done to check its safety for future development.

## 2. Materials and methods

### 2.1. Chemistry

All chemicals were commercially available. Melting points were determined on Stuart apparatus and the values given are uncorrected. The IR spectra were recorded (KBr discs) on Shimadzu IR 435 spectrophotometer, College of Pharmaceutical Sciences and Drug Manufacturing, Misr University for Science and Technology, Egypt and the values were expressed wavenumber in cm^−1^. The ^1^H-NMR and ^13 ^C-NMR spectral data were obtained on Bruker 400 MHz (Bruker Corp., Billerica, MA), Microanalytical Unit, Faculty of Pharmacy, Cairo University, Cairo, Egypt, using TMS as internal standard, and chemical shift values were recorded in ppm on *δ* scale. The mass spectra and elemental analyses were performed at the Regional Centre for Mycology and Biotechnology, Al-Azahr University, Cairo, Egypt. The reactions were monitored by precoated aluminium sheets (TLC) in benzene-acetone (7:3 v/v).

Compounds **1**^26,^^29^, **2**^29,^^30^, **3**^26,^^30^, and **5c**^31^ were prepared according to the reported procedure.

#### 2.1.1. General procedure for the preparation of 4-(4-methoxyphenyl)-2-(methylthio)-6-(substitutedpiperazin-1-yl)pyrimidine-5-carbonitrile (4a–e)

To a solution of 4-chloro-6-(4-methoxyphenyl)-2-(methylthio)pyrimidine-5-carbonitrile (**3**) (0.3 g, 1 mmol) in dry benzene; substituted piperazine derivatives (2 mmol) was added and heated under reflux for 11–14 h. The formed precipitate was filtered, washed with water (10 ml), dried, and recrystallised from acetone.

##### 2.1.1.1. 4-(4-Methoxyphenyl)-2-(methylthio)-6-(piperazin-1-yl)pyrimidine-5-carbonitrile (4a)

Yellow powder, Yield: 20%; m.p. 160–162 °C; IR (KBr, cm^−1^): 3332 (NH), 3064 (ArH), 2947, 2825, 2746 (CH aliphatic), 2200 (CN). ^1^H NMR (DMSO-d_6_-400 MHz, δ ppm): 2.09 (s, 1H, NH, exchangeable by D_2_O), 2.51 (s, 3H, S–CH_3_), 2.82 (t, 4H, CH_2_–N–CH_2_), 3.82 (t, 4H, CH_2_–N–CH_2_), 3.85 (s, 3H, OCH_3_), 7.08 (d, 2H, *J =* 8.8, ArH), 7.87 (d, 2H, *J =* 8.8, ArH), ^13 ^C NMR (DMSO-d_6_-100 MHz, δ ppm): 14.20, 45.97 (2 C), 48.70 (2 C), 55.91, 82.85, 114.27 (2 C), 118.90, 128.71, 131.63 (2 C), 162.27, 162.33, 169.54, 173.20; MS (*m/z*): 273.18 (100%), 341.33 (M^+^, 3.84%), 342.04 (M + 1, 6.48%); Anal. Calcd. For C_17_H_19_N_5_OS (341.43): C: 59.80; H: 5.61; N: 20.51. Found: C: 60.04; H: 5.83; N: 20.74.

#### 2.1.1.2. 4-(4-Methoxyphenyl)-6-(4-methylpiperazin-1-yl)-2-(methylthio)pyrimidine-5-carbonitrile (4b)

Yellowish white powder, Yield: 50%; m.p. 138–140 °C; IR (KBr, cm^−1^): 3053 (ArH), 2935, 2839, 2796 (CH aliphatic), 2204 (CN). ^1^H NMR (DMSO-d_6_-400 MHz, δ ppm): 2.22 (s, 3H, N–CH_3_), 2.45 (t, 4H, CH_2_–N–CH_2_), 2.52 (s, 3H, S–CH_3_), 3.85 (s, 3H, OCH_3_), 3.89 (t, 4H, CH_2_–N–CH_2_), 7.08 (d, 2H, *J =* 8.8, ArH), 7.88 (d, 2H, *J =* 8.8, ArH), ^13 ^C NMR (DMSO-d_6_-100 MHz, δ ppm): 14.20, 45.88, 47.11 (2 C), 54.70, 55.91 (2 C), 83.08, 114.27 (2 C), 118.84, 128.60, 131.64 (2 C), 162.30, 162.45, 169.51, 173.34; MS (*m/z*): 70.03 (100%), 355.13 (M^+^, 16.73%), 356.04 (M + 1, 2.03%); Anal. Calcd. For C_18_H_21_N_5_OS (355.46): C: 60.82; H: 5.96; N: 19.70. Found: C: 60.73; H: 6.12; N: 19.86.

##### 2.1.1.3. 4-(4-Methoxyphenyl)-2-(methylthio)-6-(4-phenylpiperazin-1-yl)pyrimidine-5-carbonitrile (4c)

Yellowish white powder, Yield: 70%; m.p. 158–160 °C; IR (KBr, cm^−1^): 3064, 3024 (ArH), 2924, 2854, 2833 (CH aliphatic), 2204 (CN). ^1^H NMR (DMSO-d_6_-400 MHz, δ ppm): 2.55 (s, 3H, S-CH_3_), 3.35 (s, 4H, CH_2_–N–CH_2_ overlapped), 3.85 (s, 3H, OCH_3_), 4.06 (s, 4H, CH_2_–N–CH_2_), 6.82 (s (br), 1H, ArH), 6.99 (d, 2H, *J =* 6.8, ArH), 7.10 (d, 2H, *J =* 8.0, ArH), 7.25 (s (br), 2H, ArH), 7.90 (d, 2H, *J =* 8.0, ArH), ^13 ^C NMR (DMSO-d_6_-100 MHz, δ ppm): 14.20, 46.98 (2 C), 48.33 (2 C), 55.95, 83.21, 114.32 (2 C), 115.93 (2 C), 118.10, 119.63, 128.79 (2 C), 129.50, 131.66 (2 C), 137.10, 150.92, 162.32, 169.50, 173.20; MS (*m/z*): 99.85 (100%), 417.33 (M^+^, 26.40%); Anal. Calcd. For C_23_H_23_N_5_OS (417.53): C: 66.16; H: 5.55; N: 16.77. Found: C: 65.89; H: 5.67; N: 16.89.

##### 2.1.1.4. 4-(4-(2-Fluorophenyl)piperazin-1-yl)-6-(4-methoxyphenyl)-2-(methylthio)pyrimidine-5-carbonitrile (4d)

Brownish powder, Yield: 70%; m.p. 142–144 °C; IR (KBr, cm^−1^): 3066, 3030 (ArH), 2960, 2922, 2839 (CH aliphatic), 2206 (CN). ^1^H NMR (DMSO-d_6_-400 MHz, δ ppm): 2.54 (s, 3H, S–CH_3_), 3.18 (s, 4H, CH_2_–N–CH_2_ overlapped), 3.86 (s, 3H, OCH_3_), 4.07 (s, 4H, CH_2_–N–CH_2_), 7.01 (t, 1H, ArH), 7.09 (d, 2H, *J =* 8.8, ArH), 7.13-7.19 (m, 3H, ArH), 7.90 (d, 2H, *J =* 8.0, ArH), ^13 ^C NMR (DMSO-d_6_-100 MHz, δ ppm): 14.26, 47.28 (2 C), 50.36 (2 C), 55.93, 83.31, 114.31 (2 C), 114.83, 116.40, 118.81, 120.03, 123.28, 125.34, 128.64 (2 C), 139.66, 154.21, 156.64, 162.35, 169.49, 173.39; MS (*m/z*): 435.31 (100%), 436.33 (M + 1, 35.22%); Anal. Calcd. For C_23_H_22_FN_5_OS (435.52): C: 63.43; H: 5.09; N: 16.08. Found: C: 63.61; H: 5.23; N: 16.27.

##### 2.1.1.5. 4-(4-Methoxyphenyl)-6-(4-(2-methoxyphenyl)piperazin-1-yl)-2-(methylthio)pyrimidine-5-carbonitrile (4e)

Brownish powder, Yield: 50%; m.p. 170–172 °C; IR (KBr, cm^−1^): 3066, 3024 (ArH), 2960, 2926, 2835 (CH aliphatic), 2200 (CN). ^1^H NMR (DMSO-d_6_-400 MHz, δ ppm): 2.54 (s, 3H, S–CH_3_), 3.12 (t, 4H, CH_2_–N–CH_2_), 3.82 (s, 3H, OCH_3_), 3.85 (s, 3H, OCH_3_), 4.06 (s, 4H, CH_2_–N–CH_2_), 6.89–6.93 (m, 2H, ArH), 6.96–6.99 (m, 2H, ArH), 7.10 (d, 2H, *J =* 8.8, ArH), 7.90 (d, 2H, *J =* 8.8, ArH), ^13 ^C NMR (DMSO-d_6_-100 MHz, δ ppm): 14.23, 47.45 (2 C), 50.39 (2 C), 55.78, 55.88, 83.04, 112.32, 114.25 (2 C), 118.70, 121.29, 123.43, 126.17, 128.75 (2 C), 131.63, 140.87, 152.43, 162.30, 162.46, 169.49, 173.38; MS (*m/z*): 105.34 (100%), 447.66 (M^+^, 43.33%); Anal. Calcd. For C_24_H_25_N_5_O_2_S (447.56): C: 64.41; H: 5.63; N: 15.65. Found: C: 64.28; H: 5.49; N: 15.86.

#### 2.1.2. General procedure for the preparation of 4-(arylamino)-6-(4-methoxyphenyl)-2-(methylthio)pyrimidine-5-carbonitrile (5a–d)

Substituted aromatic primary amine (1 mmol) was added to a mixture of 4-chloro-6-(4-methoxyphenyl)-2-(methylthio)pyrimidine-5-carbonitrile (**3**) (0.23 g, 0.8 mmol) and anhydrous potassium carbonate (0.12 g, 0.9 mmol) in absolute ethanol (15 ml). The reaction mixture was refluxed for 8–12 h. The resulting solid was filtered, washed with water (10 ml), then dried and recrystallised from ethanol.

##### 2.1.2.1. 4-(4-Methoxyphenyl)-2-(methylthio)-6-(p-tolylamino)pyrimidine-5-carbonitrile (5a)

Off white powder, Yield: 65%; m.p. 270–272 °C; IR (KBr, cm^−1^): 3292 (NH), 3049 (ArH), 2933, 2835 (CH aliphatic), 2214 (CN). ^1^H NMR (DMSO-d_6_-400 MHz, δ ppm): 2.31 (s, 3H, S–CH_3_), 2.44 (s, 3H, CH_3_), 3.86 (s, 3H, OCH_3_), 7.13 (d, 2H, *J =* 8.8, ArH), 7.18 (d, 2H, *J =* 8.0, ArH), 7.46 (d, 2H, *J =* 8.0, ArH), 7.91 (d, 2H, *J =* 8.8, ArH), 9.68 (s, 1H, NH, exchangeable by D_2_O), ^13 ^C NMR (DMSO-d_6_-100 MHz, δ ppm): 14.13, 21.00, 55.94, 83.73, 114.43 (2 C), 117.15, 124.12 (2 C), 128.45, 129.29 (2 C), 131.03 (2 C), 134.53, 135.53, 160.67, 162.21, 167.52, 174.26; MS (*m/z*): 180.18 (100%), 362.73 (M^+^, 29.50%), 364.01 (M + 2, 6.03%); Anal. Calcd. For C_20_H_18_N_4_OS (362.45): C: 66.28; H: 5.01; N: 15.46. Found: C: 66.14; H: 5.24; N: 15.72.

##### 2.1.2.2. 4-(4-Methoxyphenyl)-6-((4-methoxyphenyl)amino)-2-(methylthio)pyrimidine-5-carbonitrile (5b)

White powder, Yield: 75%; m.p. 238–240 °C; IR (KBr, cm^−1^): 3290 (NH), 3032 (ArH), 2933, 2837 (CH aliphatic), 2214 (CN). ^1^H NMR (DMSO-d_6_-400 MHz, δ ppm): 2.37 (s, 3H, S–CH_3_), 3.73 (s, 3H, OCH_3_), 3.84 (s, 3H, OCH_3_), 6.85 (d, 2H, *J =* 8.8, ArH), 7.07 (d, 2H, *J =* 8.8, ArH), 7.33 (d, 2H, *J =* 8.8, ArH), 7.85 (d, 2H, *J =* 8.8, ArH), 9.62 (s, 1H, NH, exchangeable by D_2_O), ^13 ^C NMR (DMSO-d_6_-100 MHz, δ ppm): 13.98, 55.62, 55.86, 84.28, 113.79 (2 C), 114.19 (2 C), 118.34, 125.55 (2 C), 129.26, 130.74 (2 C), 135.50, 155.88, 160.02, 161.73, 166.83, 173.11; MS (*m/z*): 180.16 (100%), 387 (M^+^, 20.50%); Anal. Calcd. For C_20_H_18_N_4_O_2_S (378.45): C: 63.47; H: 4.79; N: 14.80. Found: C: 63.19; H: 4.95; N: 15.07.

##### 2.1.2.3. 4-((4-Bromophenyl)amino)-6-(4-methoxyphenyl)-2-(methylthio)pyrimidine-5-carbonitrile (5d)

White powder, Yield: 20%; m.p. charring >300 °C; IR (KBr, cm^−1^): 3292 (NH), 3116 (ArH), 2895 (CH aliphatic), 2216 (CN). ^1^H NMR (DMSO-d_6_-400 MHz, δ ppm): 2.34 (s, 3H, S–CH_3_), 3.83 (s, 3H, OCH_3_), 7.05 (d, 2H, *J =* 8.8, ArH), 7.20 (d, 2H, *J =* 8.4, ArH), 7.32 (d, 2H, *J =* 8.8, ArH), 7.82 (d, 2H, *J =* 8.4, ArH), 9.85 (s, 1H, NH, exchangeable by D_2_O), ^13 ^C NMR (DMSO-d_6_-100 MHz, δ ppm): 13.90, 55.83, 84.50, 114.05, 114.19 (2 C), 118.50, 125.60 (2 C), 128.22 (2 C), 130.64 (2 C), 139.22, 157.70, 161.22, 162.53, 166.60, 180.12; MS (*m/z*): 181.64 (100%), 427.71 (M^+^, 12.99%); Anal. Calcd. For C_19_H_15_BrN_4_OS (427.32): C: 53.40; H: 3.54; N: 13.11. Found: C: 53.62; H: 3.78; N: 13.29.

##### 2.1.2.4. 4-Hydrazineyl-6-(4-methoxyphenyl)-2-(methylthio)pyrimidine-5-carbonitrile (6)

Hydrazine hydrate 99% (0.45 ml, 9 mmol) was added dropwise to a solution of chloro compound **3** (0.9 g, 3 mmol) in methanol (10 ml). The reaction mixture was stirred for 5 h at room temperature then the precipitate was filtered, washed, and crystallised from methanol.

Pale yellow powder, Yield: 80%; m.p. 270–272 °C; IR (KBr, cm^−1^): 3346, 3255 (NH, NH_2_), 3032 (ArH), 2933, 2839 (CH aliphatic), 2202 (CN). ^1^H NMR (DMSO-d_6_-400 MHz, δ ppm): 2.56 (s, 3H, S–CH_3_), 3.86 (s, 3H, OCH_3_), 5.04 (s, 2H, NH_2_, exchangeable by D_2_O), 7.14 (d, 2H, *J =* 8.4, ArH), 7.88 (d, 2H, *J =* 8.4, ArH), 12.53 (s, 1H, NH, exchangeable by D_2_O), MS (*m/z*): 287.09 (M^+^, 100%), 288.03 (M + 1, 14.58%); Anal. Calcd. For C_13_H_13_N_5_OS (287.34): C: 54.34; H: 4.56; N: 24.37. Found: C: 54.62; H: 4.63; N: 24.60.

#### 2.1.3. General procedure for the preparation of 4-(2-arylidenehydrazineyl)-6-(4-methoxyphenyl)-2-(methylthio)pyrimidine-5-carbonitrile (7a–f)

A mixture of hydrazinyl compound **6** (0.5 g, 1.7 mmol), glacial acetic acid (2 ml) and the appropriate aldehydes (1.7 mmol) in absolute ethanol (20 ml) was heated under reflux for 10–12 h. The precipitate was filtered and crystallised from ethanol.

##### 2.1.3.1. 4–(4-Methoxyphenyl)-6-(2-(4-methylbenzylidene)hydrazineyl)-2-(methylthio)pyrimidine-5-carbonitrile (7a)

White powder, Yield: 45%; m.p. 246–248 °C; IR (KBr, cm^−1^): 3263 (NH), 3118 (N = CH), 3055 (ArH), 2922, 2837 (CH aliphatic), 2210 (CN). ^1^H NMR (DMSO-d_6_-400 MHz, δ ppm): 2.34 (s, 3H, CH_3_), 2.57 (s, 3H, S–CH_3_), 3.86 (s, 3H, OCH_3_), 7.10 (d, 2H, *J =* 8.8, ArH), 7.25 (d, 2H, *J =* 8.0, ArH), 7.72 (d, 2H, *J =* 8.0, ArH), 7.90 (d, 2H, *J =* 8.8, ArH), 8.17 (s, 1H, N = CH), 12.16 (s, 1H, NH, exchangeable by D_2_O), ^13 ^C NMR (DMSO-d_6_-100 MHz, δ ppm): 14.05, 21.52, 55.88, 82.16, 114.13 (2 C), 118.05, 127.79 (2 C), 128.81, 129.85 (2 C), 131.51 (2 C), 131.89, 140.43, 147.05, 160.21, 162.03, 173.13, 177.50, MS (*m/z*): 272.21 (100%), 389.67 (M^+^, 7.48%); Anal. Calcd. For C_21_H_19_N_5_OS (389.48): C: 64.76; H: 4.92; N: 17.98. Found: C: 64.89; H: 5.13; N: 18.21.

##### 2.1.3.2. 4-(2-(4-Methoxybenzylidene)hydrazineyl)-6-(4-methoxyphenyl)-2-(methylthio)pyrimidine-5-carbonitrile (7b)

Yellowish white powder, Yield: 35%; m.p. 244–246 °C; IR (KBr, cm^−1^): 3257 (NH), 3124 (N = CH), 3010 (ArH), 2922, 2839 (CH aliphatic), 2214 (CN). ^1^H NMR (DMSO-d_6_-400 MHz, δ ppm): 2.56 (s, 3H, S–CH_3_), 3.81 (s, 3H, OCH_3_), 3.86 (s, 3H, OCH_3_), 7.00 (d, 2H, *J =* 8.8, ArH), 7.10 (d, 2H, *J =* 8.8, ArH), 7.78 (d, 2H, *J =* 8.8, ArH), 7.98 (d, 2H, *J =* 8.8, ArH), 8.14 (s, 1H, N = CH), 12.12 (s, 1H, NH, exchangeable by D_2_O), ^13 ^C NMR (DMSO-d_6_-100 MHz, δ ppm): 14.04, 55.76, 55.89, 82.00, 114.14 (2 C), 114.75 (2 C), 118.50, 127.31, 128.84, 129.48 (2 C), 131.50 (2 C), 146.05, 160.10, 161.29, 162.03, 166.50, 173.38, MS (*m/z*): 223.54 (100%), 405.24 (M^+^, 18.88%); Anal. Calcd. For C_21_H_19_N_5_O_2_S (405.48): C: 62.21; H: 4.72; N: 17.27. Found: C: 62.40; H: 4.86; N: 17.53.

##### 2.1.3.3. 4-(2-(4-Chlorobenzylidene)hydrazineyl)-6-(4-methoxyphenyl)-2-(methylthio)pyrimidine-5-carbonitrile (7c)

White powder, Yield: 70%; m.p. 266–268 °C; IR (KBr, cm^−1^): 3265 (NH), 3130 (N = CH), 3026 (ArH), 2922, 2839 (CH aliphatic), 2212 (CN). ^1^H NMR (DMSO-d_6_-400 MHz, δ ppm): 2.57 (s, 3H, S–CH_3_), 3.86 (s, 3H, OCH_3_), 7.11 (d, 2H, *J =* 8.4, ArH), 7.51 (d, 2H, *J =* 8.0, ArH), 7.86 (d, 2H, *J =* 8.0, ArH), 7.91 (d, 2H, *J =* 8.4, ArH), 8.19 (s, 1H, N = CH), 12.29 (s, 1H, NH, exchangeable by D_2_O), ^13 ^C NMR (DMSO-d_6_-100 MHz, δ ppm): 14.08, 55.88, 82.34, 114.12 (2 C), 118.18, 128.75, 129.29 (2 C), 129.33 (2 C), 131.54 (2 C), 133.69, 134.91, 145.50, 160.33, 162.08, 170.80, 173.48, MS (*m/z*): 229.61 (100%), 409.02 (M^+^, 25.26%); Anal. Calcd. For C_20_H_16_ClN_5_OS (409.89): C: 58.61; H: 3.93; N: 17.09. Found: C: 58.82; H: 4.09; N: 17.26.

##### 2.1.3.4. 4-(2-(4-Bromobenzylidene)hydrazineyl)-6-(4-methoxyphenyl)-2-(methylthio)pyrimidine-5-carbonitrile (7d)

Yellowish white powder, Yield: 50%; m.p. 246–248 °C; IR (KBr, cm^−1^): 3259 (NH), 3118 (N = CH), 3022 (ArH), 2920, 2839 (CH aliphatic), 2210 (CN). ^1^H NMR (DMSO-d_6_-400 MHz, δ ppm): 2.57 (s, 3H, S–CH_3_), 3.86 (s, 3H, OCH_3_), 7.11 (d, 2H, *J =* 8.8, ArH), 7.65 (d, 2H, *J =* 8.8, ArH), 7.78 (d, 2H, *J =* 8.8, ArH), 7.91 (d, 2H, *J =* 8.8, ArH), 8.17 (s, 1H, N = CH), 12.28 (s, 1H, NH, exchangeable by D_2_O), ^13 ^C NMR (DMSO-d_6_-100 MHz, δ ppm): 14.09, 55.91, 82.38, 114.17 (2 C), 118.30, 123.77, 128.75 (2 C), 129.58 (2 C), 131.55 (2 C), 132.25, 134.03, 145.20, 160.37, 162.11, 170.95, 176.05, MS (*m/z*): 378.38 (100%), 454.79 (M + 1, 20.96%); Anal. Calcd. For C_20_H_16_BrN_5_OS (453.03): C: 52.87; H: 3.55; N: 15.41. Found: C: 53.14; H: 3.67; N: 15.68.

##### 2.1.3.5. 4–(4-Methoxyphenyl)-2-(methylthio)-6-(2-(4-nitrobenzylidene)hydrazineyl)pyrimidine-5-carbonitrile (7e)

Yellow powder, Yield: 70%; m.p. 248–250 °C; IR (KBr, cm^−1^): 3292 (NH), 3099 (N = CH), 3039 (ArH), 2935, 2839 (CH aliphatic), 2210 (CN). ^1^H NMR (DMSO-d_6_-400 MHz, δ ppm): 2.56 (s, 3H, S–CH_3_), 3.86 (s, 3H, OCH_3_), 7.10 (d, 2H, *J =* 8.0, ArH), 7.91 (d, 2H, *J =* 8.0, ArH), 8.03 (d, 2H, *J =* 8.0, ArH), 8.23 (d, 2H, *J =* 8.0, ArH), 8.80 (s, 1H, N = CH), 12.46 (s, 1H, NH, exchangeable by D_2_O), ^13 ^C NMR (DMSO-d_6_-100 MHz, δ ppm): 14.10, 55.89, 82.63, 114.12 (2 C), 118.30, 124.30 (2 C), 128.44 (2 C), 129.98, 131.57 (2 C), 140.99, 143.17, 147.97, 160.39, 162.14, 166.50, 173.62, MS (*m/z*): 150.07 (100%), 420.90 (M^+^, 24.51%); Anal. Calcd. For C_20_H_16_N_6_O_3_S (420.45): C: 57.13; H: 3.84; N: 19.99. Found: C: 57.41; H: 3.98; N: 19.86.

##### 2.1.3.6. 4-(4-Methoxyphenyl)-2-(methylthio)-6-(2-(3,4,5-trimethoxybenzylidene)hydrazineyl)pyrimidine-5-carbonitrile (7f)

Yellow powder, Yield: 30%; m.p. 222–224 °C; IR (KBr, cm^−1^): 3286 (NH), 3151 (N = CH), 3134 (ArH), 2935, 2837 (CH aliphatic), 2210 (CN). ^1^H NMR (DMSO-d_6_-400 MHz, δ ppm): 2.61 (s, 3H, S–CH_3_), 3.75 (s, 3H, OCH_3_), 3.82 (s, 3H, OCH_3_), 3.85 (s, 6H, (OCH_3_)_2_), 7.08 (d, 2H, *J =* 8.0, ArH), 7.27 (s, 2H, ArH), 8.24 (d, 2H, *J =* 8.0, ArH), 8.83 (s, 1H, N = CH), 13,71 (s, 1H, NH, exchangeable by D_2_O), ^13 ^C NMR (DMSO-d_6_-100 MHz, δ ppm): 14.22, 55.78, 56.35 (2 C), 60.65, 103.00, 106.73, 107.15, 113.73 (2 C), 114.63, 128.74, 131.28, 133.04 (2 C), 141.41, 143.26, 153.60 (2 C), 161.21, 162.14, 168.95, 192.33, MS (*m/z*): 161.88 (100%), 465.90 (M^+^, 3.13%); Anal. Calcd. For C_23_H_23_N_5_O_4_S (465.53): C: 59.34; H: 4.98; N: 15.04. Found: C: 59.48; H: 5.12; N: 15.27.

### 2.2. Biological activity

#### 2.2.1. MTT colorimetric assay

Cytotoxicity of our sixteen innovative compounds **4(a–e), 5(a–d)**, **6**, and **7(a–f)** together with control was assessed by means of *in-vitro* toxicology assay kit, MTT based (Sigma Aldrich, St. Louis, MO) against MCF-7 and K562 cell lines incubated in 96 microwell plates and proceeded as reported^32^^,^^33^. MTT (3-[4,5-dimethylthiazol-2-yl]-2,5-diphenyl tetrazolium bromide) method measures the activity of living cells through mitochondrial dehydrogenase. Mitochondrial dehydrogenases of viable cells cleave the tetrazolium ring, giving purple formazan crystals that are dissolved in acidified isopropanol and the resulting purple solution is measured spectrophotometrically. The change in cell number is proportional to the intensity of purple colour produced and the degree of cytotoxicity caused by the test compounds. The absorbance at a wavelength of 570 nm was measured using (ROBONIK P2000 spectrophotometer) and the background absorbance of multi-well plates at 690 nm then the absorbance at 690 nm was subtracted from the 570 nm measurement. Five concentrations from each test compound ranging from (0.04 to 100 µM) were prepared and the experiments were done in triplicate. IC_50_ values (the concentration required for 50% inhibition) were calculated using dose-response curves and linear regression equation.

#### 2.2.2. P*I3K enzyme inhibition assay*

The inhibition activities of **4d** and **7f** against PI3K enzyme were detected using PI3K assay kits and ADP-Glo kinase assay as a detection buffer (BPS Bioscience, San Diego, CA). According to the manufacturer’s instructions, PI3K enzyme was diluted with 2.5x Kinase assay buffer to obtain 4 ng/µl. The assay was performed in 96 well plates as follows: 5 µl of PIP2 substrate was added to each well followed by 5 ul from each inhibitor except for the positive control and blanks where the inhibitor buffers were added instead. Of 5 ul of 12.5 µM ATP was added. The reactions were initiated by adding 10 µl of diluted PI3K enzyme to the positive control and inhibitors wells. Plate was carefully shaked and was incubated at 30 °C for 40 min. After the 40 min incubation, 25 µl of ADP-Glo reagent were added to each well. The plate was covered with aluminium foil and incubated at room temperature for another 45 min. Of 50 µl of Kinase Detection reagent was added to each well. Plate was covered with aluminium foil and incubated at room temperature for extra 30 min. Luminescence was measured using microplate reader. Four concentrations from the two tested compounds ranged from (0.1 to 100 µM) were prepared and the experiment was repeated in triplicate. The results were expressed in the form of IC_50_ values (the concentration required for 50% inhibition). IC_50_ values were calculated using dose-response curves and linear regression equation. LY294002 compound was used as the positive control. The inhibition/dose-response plots of the tested compounds and the control are listed in the Supplementary Materials.

#### 2.2.3. AKT enzyme inhibition assay

AKT activity was measured in cell lysates for the tested compounds (**4d** and **7f**) using enzyme-linked immuno-absorbent assay (ELISA) (Abcam, Cat No. ab139436, Cambridge, UK) according to the manufacturer’s protocol. In brief, cell lysates were collected after treatment with the tested compounds using lysis buffer and centrifuged at 13,000 rpm for 15 min to obtain the cytosolic fraction. Samples were soaked with the kinase diluted buffer and the reaction was initiated by adding 10 µl of diluted ATP to each well, except blanks. Plate was incubated at 30 °C for up to 90 min covered with an adhesive plate sealer with gentle, shaking every 20 min. The reaction was stopped by emptying contents of each well. Of 40 µl of the phospho-specific substrate antibody was added except blank then incubated incubate at room temperature for 60 min, with shaking every 20 min. The liquid was aspirated and the wells washed three times with 1X washing buffer. After the last wash, liquid was aspirated and 40 µl of the diluted Anti-Rabbit IgG: HRP conjugate was added except blank, and incubated at room temperature for 30 min with shaking every 10 min. Plate was washed as described before. Of 60 µl of the TMB Substrate was added to each well and incubated at room temperature for 30–60 min. The reaction was stopped by adding 20 µl of the stop solution to each well. Absorbance was measured at 450 nm. Four concentrations from the tested compounds were prepared ranged from 0.1 to 100 µM and the assay was done in triplicates. The results were expressed in the form of IC_50_ values (the concentration required for 50% inhibition) using dose-response curves and linear regression equation. LY2780301 compound was used as the positive control. The inhibition/dose-response plots of the tested compounds and the control are listed in the Supplementary Materials.

#### 2.2.4. Flowcytometric analysis and apoptosis

Cell cycle analysis and apoptosis induction of the most potent compound **7f** was tested against leukaemia cell line (K562).

##### 2.2.4.1. Cell cycle analysis

Cell cycle analysis was determined by propidium iodide (PI) flow cytometry kit/BD. K562 cells were treated by compound **7f** at its IC_50_ concentration. Then cells were washed in PBS, fixed in cold 70% ethanol for 30 min, washed twice in PBS, and centrifuged to remove supernatant, after that, cells were treated with 0.1 mg/ml ribonuclease, stained with 0.05 mg/ml PI. The results were analysed by flow cytometry using FACS calibre and cell cycle distribution were calculated and accomplished as reported^26^^,^^34^^,^^35^.

##### 2.2.4.2. Annexin-V-FITC apoptosis assay

Apoptosis induction was assayed by staining K562 cells with Annexin-V (fluorescein isothiocyanate) and the counterstaining PI using the Annexin-V-FITC/PI apoptosis detection kit (BD Biosciences, San Diego, CA) according to the manufacturer’s instructions. Cells were exposed to the tested compound for 48 h. After that, the cells were collected by trypsinisation and 0.5 × 10^6^ cells and washed twice with phosphate-buffered saline (PBS) followed by staining with 5 µl Annexin-V-FITC and 5 µl PI in 1 × binding buffer for 15 min at room temperature in the dark. FACS Calibre flow cytometer (BD Biosciences, San Diego, CA)^26^^,^^36^^,^^37^ was used for analyses.

#### 2.2.5. Determination of the effect of 7f on active caspase 3

The concentration of human active caspase 3 was measured using *in-vitro* gen (catalogue KH 01091) ELISA kit. Of 100 µl of the standard diluents buffer were added to the zero standard wells. Then 100 µl of standard and controls were added to microlitre wells. Wells were covered and incubated for 2 h at room temp. After wells were decanted, 100 µl of active caspase 3 detection antibody solution were added into each well. Plates covered and incubated for 1 h at room temp. Then wells were decanted again and 100 µl of anti-rabbit IgG HRP were added to each well. Wells were covered again and incubated for 30 min. wells were decanted and 100 µl of stabilised chromogen were added. The wells began to turn blue and then incubated for 30 min. Finally, a stop solution was added and the colour in the wells changed from blue to yellow. The plate was read using ROBONIK P2000 ELISA reader at 450 nm and standard curve was obtained^38^.

#### 2.2.6. Western blot analysis

K562 cells were treated with the most promising compound (**7f**), for 48 h. Protein from control and treated cells was harvested and the concentration was measured *via* Bradford protein assay (Bio-Rad, Hercules, CA). The samples were loaded and separated by sodium dodecyl sulphate (SDS)-polyacrylamide gel electrophoresis and then transferred onto a polyvinylidene difluoride (PVDF) membrane. Western blot analysis was performed according to previously described^39^.

#### 2.2.7. *In-vivo* acute toxicity study

Five female rats (180–200 g) were used in this test. The animals were fasted for 24 h prior to administration of the test compound. The compound was administered in a dose of 2 g/kg body weight, po . Animals are observed individually and the mortalities were recorded at least once during the first 30 min after dosing, periodically during the first 24 h and daily afterwards, for a total of 14 d^40^. All animals received humane care and experimental procedures were carried out in strict accordance with the health and care guidelines for experimental animals. All experimental operations performed on rats were approved by the Animal Experiment Ethics Committee of Heliopolis University (Approval number HU.REC.A0.23-2021).

### 2.3. Molecular modelling

Molecular modelling study was accomplished using Discovery Studio software version 4.1 (Accelrys, Inc., San Diego, CA). The target compounds were drawn on ChemDraw Professional version 16.0 (PerkinElmer Inc, Waltham, MA) and subjected to energy minimisation using CHARMm-Force Field. The X-ray crystal structure of each enzyme was retrieved from the protein data bank (http://www.rscb.org/pdb)^41^^,^^42^. First, the enzyme protein was prepared for docking by removing the unwanted chain and water molecules then protonated, and missing amino acids were fixed. Second, the minimisation step was carried after applying fixed atom constraints on heavy atoms to keep the 3D structure of the protein. After that, the binding pocket was identified using the co-crystalised ligand and the validation step was performed. Finally, the docking of the prepared new ligands into the 3D structure of the protein was carried out adopting flexible ligand-rigid receptor docking in the CDOCKER protocol. The best 10 docked conformers for each ligand were retained and their CDOCKER interaction scores were studied to get the pose which has the closest binding pattern like that of the bioactive conformation of the lead molecule.

## 3. Results and discussion

### 3.1. Chemistry

**Scheme 1.**
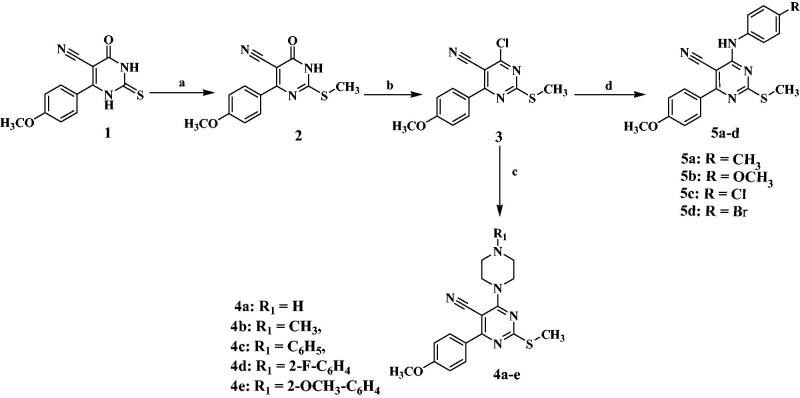
Reagents and Conditions: a) CH_3_I/anhydrous K_2_CO_3_/DMF/stirring 3 h 0–5 °C; b) POCl_3_/reflux 5 h; c) piperazine derivatives/dry benzene/reflux 11–14 h; d) aniline derivatives/K_2_CO_3_/abs. ethanol/reflux 8–12 h.

**Scheme 2.**
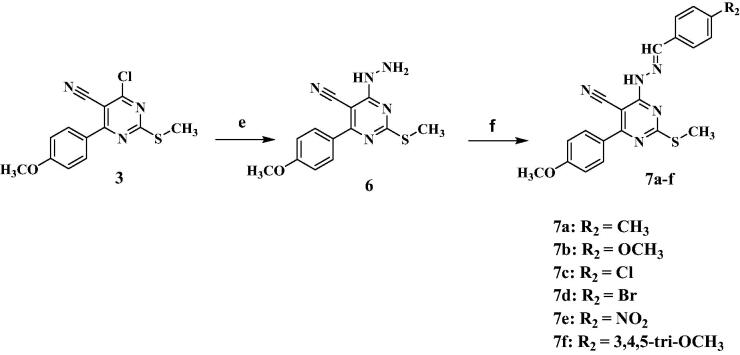
Reagents and conditions: e) NH_2_NH_2_/methanol/stirring 5 h at room temperature; f) aromatic aldehyde derivatives/glacial acetic acid/absolute ethanol/reflux 10–12 h.

The proposed synthesising approach is outlined in Schemes 1 and 2 where the starting compound **(1)**^26,^^29^ and the intermediates **(2)**^29^^,^^30^and **(3)**^26,^^30^ were prepared as reported and also derivative **(5c)** was prepared as reported^31^. First, the one-pot three-component Bigenilli reaction was adopted for the synthesis of the starting pyrimidinone-5-carbonitrile (**1)**. Regioselective methylation of **(1)** with methyl iodide in dimethylformamide at 0 °C in the presence of anhydrous potassium carbonate yielded 2-metylthio-4-(4-methoxyphenyl)-6-oxo-1,6-dihydropyrimidine-5-carbonitrile **(2)**. Refluxing intermediate **(2)** with phosphorusoxychloride afforded the key intermediate 4-chloro-6–(4-methoxyphenyl)-2-(methylthio)pyrimidine-5-carbonitrile **(3)** which was confirmed by the disappearance of NH and C = O amide bands from the IR spectrum and the disappearance of NH peak in ^1^H-NMR spectrum that confirms chlorination step. The key intermediate **(3)** was then refluxed with different piperazine derivatives in dry benzene to afford 4-(4-methoxyphenyl)-2-(methylthio)-6-(substitutedpiperazin-1-yl)pyrimidine-5-carbonitrile **(4a–e).** Their ^1^H-NMR spectra revealed two characteristic signals around δ 2.45–3.12 ppm and δ 3.82–4.07 ppm corresponding to piperazine protons. Moreover, Aniline analogues **(5a–d)** were obtained through refluxing the intermediate **3** in presence of anhydrous potassium carbonate and absolute ethanol with different aniline derivatives. The ^1^H NMR spectra of **(5a–d)** presented an exchangeable singlet peak around δ 9.62–9.85 ppm due to the presence of NH protons. In addition, the appearance of aromatic protons in the aromatic range as detailed in the experimental part alongside the appearance of the NH band around 3290–3292 cm^−1^ in IR spectra. The chloro compound **(3)** was reacted with hydrazine hydrate 99% in methanol at room temperature providing the second intermediate 4-hydrazineyl-6-(4-methoxyphenyl)-2-(methylthio)pyrimidine-5-carbonitrile **(6)** which then was refluxed with the appropriate aldehydes in absolute ethanol and catalytic amounts of glacial acetic acid to give the corresponding Schiff’s bases derivatives 4-(2-arylidenehydrazineyl)-6-(4-methoxyphenyl)-2-(methylthio)pyrimidine-5-carbonitrile (**7a–f)**. Their structures were confirmed by the disappearance of NH_2_ forked peak of compound **6** in the IR spectra while the NH band was still present. Additionally, ^1^H-NMR spectra showed a singlet signal of NH proton around δ 12.12–13.71 ppm exchangeable with D_2_O and another singlet signal around δ 8.14-8.83 ppm assigned to N = CH proton.

### 3.2. Biological activity

#### 3.2.1. *In-vitro* cytotoxic activity assay against breast cancer cell line (MCF-7) and leukaemia cell line (K562)

The newly synthesised compounds **4a–e**, **6, 5a–d**, and **7a–f** were assessed for their cytotoxic activity against MCF-7 and K562 cell lines with the MTT (3-(4,5-dimethylthiazol-2-yl)-2,5- diphenyltetrazolium bromide) colorimetric assay^32^^,^^33^ using Staurosporine as the positive control. The cytotoxic efficacy of these compounds was expressed as IC_50_ value representing the concentration of the compound required to produce a 50% inhibition of cell growth after 48 h of incubation with the tested compounds as shown in Table 1.

**Table 1.** IC_50_ values of the new compounds against breast cancer (MCF-7) and leukaemia (K562) cell lines.

	Cytotoxicity in IC_50_ (uM)^a^	
Compounds	MCF-7	K562
Staurosporine	9.51 ± 0.52	11.58 ± 0.55
**4a**	2.54 ± 0.14***	7.97 ± 0.38**
**4b**	56.28 ± 3.06**	93.61 ± 4.44***
**4c**	6.64 ± 0.36**	3.86 ± 0.18***
**4d**	0.80 ± 0.25***	1.06 ± 0.05***
**4e**	15.57 ± 0.85**	34.81 ± 1.65***
**5a**	21.33 ± 1.16***	26.32 ± 1.25***
**5b**	125.4 ± 6.82**	46.31 ± 2.20***
**5c**	46.89 ± 2.55**	5.82 ± 0.28***
**5d**	15.66 ± 0.85**	5.52 ± 0.26***
**6**	3.491 ± 0.19***	6.67 ± 0.32***
**7a**	30.49 ± 1.66***	22.79 ± 1.08***
**7b**	22.85 ± 1.24***	16.81 ± 0.80**
**7c**	7.42 ± 0.40**	3.76 ± 0.18***
**7d**	55.97 ± 3.05**	44.33 ± 2.10***
**7e**	7.11 ± 0.39**	5.36 ± 0.25***
**7f**	3.22 ± 0.18***	2.62 ± 0.12***

^a^IC_50_ values are the mean ± SD of three separate experiments.

**p < .01, and ***p < .001.

The IC_50_ values revealed that most of the newly developed pyrimidine derivatives demonstrated high cytotoxic activity on both cell lines compared to the reference compound Staurosporine (9.51 ± 0.52 uM on MCF-7 and 11.58 ± 0.55 uM on K562) with better activity on leukaemia cell line than on the breast cancer cell line.

In the piperazine series (**4a–e**), the most active compound was the *o*-F-phenyl derivative **4d** with IC_50_ value of 0.80 ± 0.25 uM on MCF-7 and 1.06 + 0.05 uM on K562 while the unsubstituted-phenylpiperazine derivative **4c** has lower activity with IC_50_ value of 6.64 ± 0.36 uM on MCF-7 and 3.86 ± 0.18 uM on K562. Compound **4a** the unsubstituted piperazine derivative gave better activity on the MCF-7 cell line with IC_50_ value of 2.54 ± 0.14 uM while on K562 cell line it gave 7.97 ± 0.38 uM. The 2-methoxyphenyl piperazine **4e** and the 4-methyl piperazine derivative **4b** were the least active against MCF-7 and K562 cell lines. These indicate the importance of an aromatic moiety at the 4-position of the piperazine ring.

In the aniline series (**5a–d**), the activity was decreased compared to the piperazine series. The two promising compounds in this group are the 4-chloro and 4-bromo-aniline derivatives **5c** and **5d** with IC_50_ value of 5.82 ± 0.28 and 5.52 ± 0.26 uM on K562 cell line, respectively. These results display the importance of the presence of an aromatic ring substituted with a halogen.

Moreover, by increasing the linker length to be three atoms utilising the hydrazono linker between the aromatic moiety and the pyrimidine-5-carbonitrile scaffold in **7a–f** series enhanced the cytotoxic activity again especially on leukaemia K562 cell line. Compound **7f** manifested excellent cytotoxic activity with IC_50_ value of 3.22 ± 0.18 uM on MCF-7 and 2.62 ± 0.12 uM on K562 cell lines. Also, compounds **7c** and **7e** exhibited remarkable cytotoxic activity with IC_50_ values of 3.76 ± 0.18 and 5.36 ± 0.25 uM, respectively, on the K562 cell line indicating the positive effect of electron withdrawing groups on the cytotoxic activity.

In conclusion, it was found that Schiff’s derivatives **7a–f** and piperazine derivatives **4a–e** series have better cytotoxic activity over aniline derivatives **5a–d** which may be attributed to the presence of a linker (3 or 4 atoms in length) between the pyrimidine scaffold and the aromatic terminal that allow better interaction with their targets.

Alongside, the cytotoxicity of the most active compounds **4d** and **7f** were evaluated on a normal fibroblast cell line (WI-38) to judge their safety profile^43^. The results shown in Table 2 clarified that both candidate **4d** and **7f** have better selective toxicity towards cancer cells with selectivity index (SI = 109.72 and 13.29, respectively) than the reference drug Staurosporine with SI = 1.97. This suggested that the new compounds exhibited a good safety profile.

**Table 2.** IC_50_ values of the most active compounds against non-tumorigenic cell line.

	Cytotoxicity in IC_50_ (uM)		
Compounds	K562	WI-38	SI
**4d**	1.06 ± 0.05	116.3 ± 7.03	109.72
**7f**	2.62 ± 0.12	34.83 ± 2.11	13.29
Staurosporine	11.58 ± 0.55	22.8 ± 1.38	1.97

#### 3.2.2. PI3K/AKT enzyme inhibition assays

In order to investigate the molecular mechanism by which the most active compounds **4d** and **7f** produce their potential antiproliferative action, enzyme inhibition assays were done on PI3Kγ, PI3Kδ, and AKT-1 isoforms known for their effects on cell survival and proliferation. Compound **7f** exhibited a significant inhibitory activity value on the three tested enzymes compared to the reference compound LY294002 as depicted in Table 3 with *p* < .01, respectively. Compound **7f** was more active to PI3Kδ isoform than PI3K**γ** isoform which is known for its overexpression in leukaemia, in addition to its excellent inhibitory activity on AKT enzyme. These results support the objectives of designing dual PI3K/AKT inhibitors to overcome the compensatory resistance developed with single enzyme-acting inhibitors. For compound **4d**, it gave less inhibitory activity on PI3K isoforms and moderate inhibition on AKT-1. Thus, compound **7f** was chosen to further explore its effects on apoptosis as an endpoint for inhibiting this signal transduction axis.

**Table 3.** IC_50_ values of PI3Kγ, PI3Kδ, and AKT Inhibition of compounds **4d** and **7f**.

		IC_50_ (uM)^a**^	
Compounds	PI3Kγ	PI3Kδ	AKT
**4d**	35.10 ± 1.82	23.90 ± 1.20	7.67 ± 0.40
**7f**	6.99 ± 0.36	4.01 ± 0.55	3.36 ± 0.17
**LY294002**	15.7 ± 0.81	13.4 ± 0.67	–
**LY2780301**	–	–	4.62 ± 0.24

^a^IC_50_ values are the mean ± SD of three separate experiments.

**p < .01.

#### 3.2.3. Cell cycle analysis and apoptosis induction

**Figure 5.**
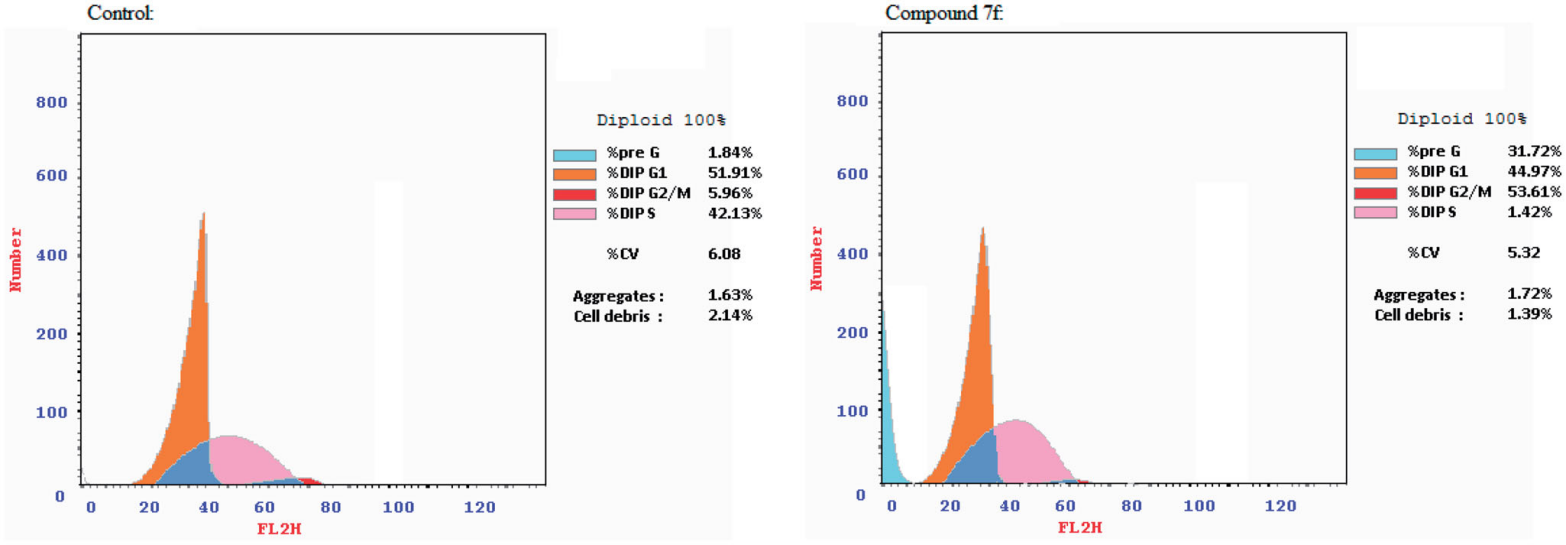
The distribution of cells in cell cycle phases after treatment with the tested compound **7f**.

As reported in the literature, inhibition of PI3K/AKT pathway affects the cell survival and proliferation^8^^,^^9^. Thus, to investigate the effect of compound **7f** in PI3K/Akt dual inhibition on cell cycle progression, cell cycle analysis was performed by flowcytometric analysis as reported^34^^,^^35^ on K562 cells treated with compound **7f.** The outcomes presented in Table 4 indicated the interference with the cell cycle distribution, following **7f** treatment, where there was a marked increase in the cells in pre-G1 phase by nearly 17-fold as compared to the control. In addition, it showed an increase in S-phase by almost 1.3-fold as displayed in Figure 5. These results demonstrated the antiproliferative effect of compound **7f** as evidenced by promoting S-phase cell cycle arrest and apoptosis induction.

**Table 4.** Cell cycle analysis of leukaemia K562 cells after treatment with the most active compound **7f**.

DNA content %					
Compound	%G0-G1	%S	%G2/M	%Pre-G1	Comment
**7f**	44.97	53.61	1.42	31.72	Cell growth arrest at S phase
**Control K562**	51.91	42.13	5.96	1.84	–

**Figure 6.**
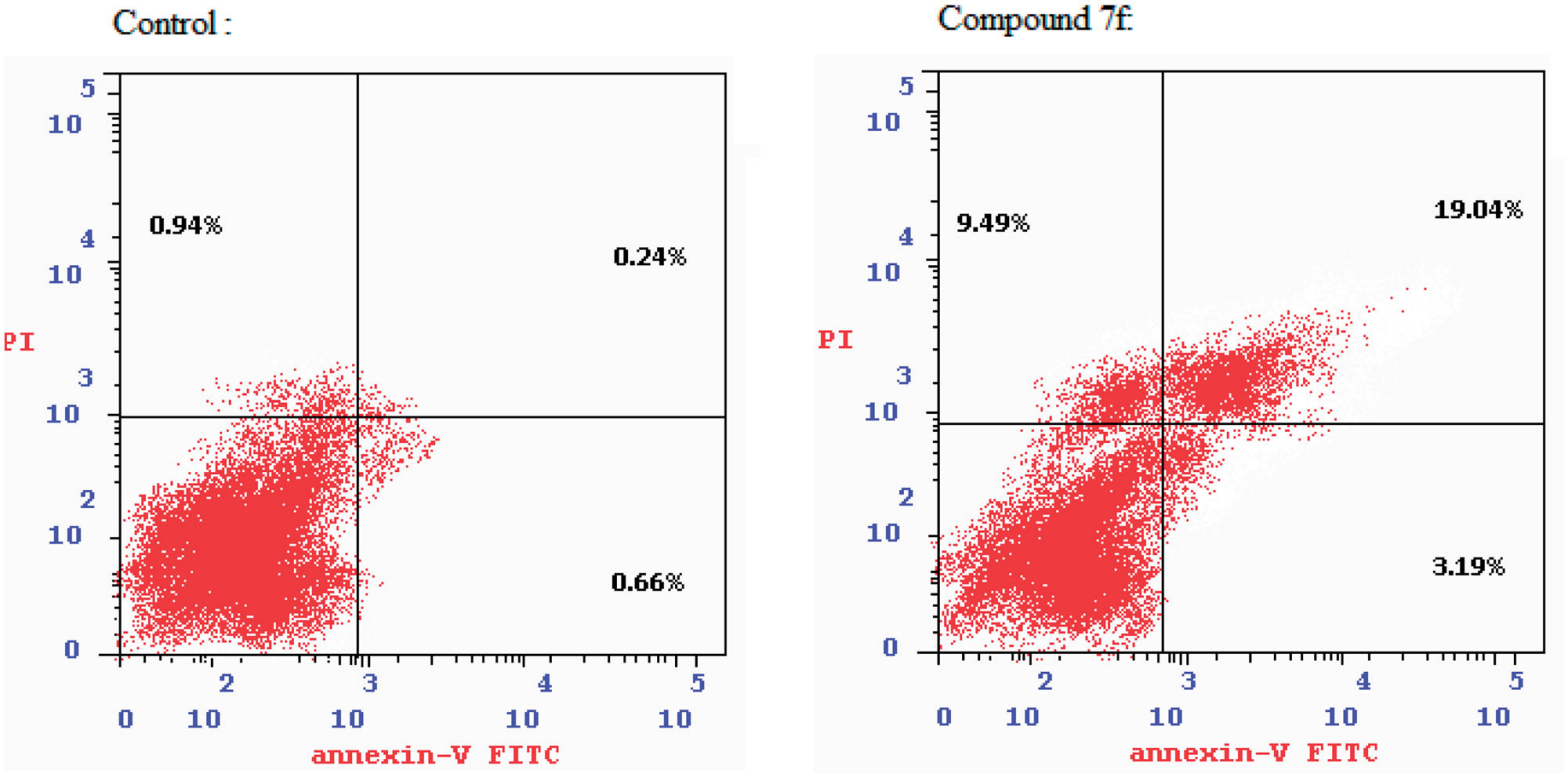
Effect of the active compound **7f** on inducing apoptosis in Leukaemia K562 cell line.

Consequently, the pro-apoptotic activity of compound **7f** was explored^36^^,^^37^ as shown in Table 5. Data of the current research highlighted that treating Leukaemia K562 cells for 48 h with the compound **7f** caused an increase in the percentage of Annexin-V positive apoptotic cells both in the early and late stage which represents a total significant 17-fold increase compared to the control. This finding suggests that the reported cytotoxic activity of the trimethoxy derivative **7f** is attributed to apoptosis induction; as demonstrated in Figure 6.

**Table 5.** Percentage of apoptotic cells in the Annexin-V-FITC experiment.

	Apoptosis%			
Compound	Total	Early	Late	Necrosis
**7f**	31.72	3.19	19.04	9.49
**Control K562**	1.84	0.66	0.24	0.94

**Table 6.** Protein expression of downstream effectors of PI3K/AKT.

Western blot [OD]						
Compound	Cyclin D1	AKT	p-AKT	PI3K	p-PI3K	NFkb
**7f**	0.383	0.402	0.316	0.219	0.281	0.402
**Control**	0.849	0.744	0.692	0.881	0.639	0.719

#### 3.2.4. Caspase 3 enzyme assay using ELISA in K562

**Figure 7.**
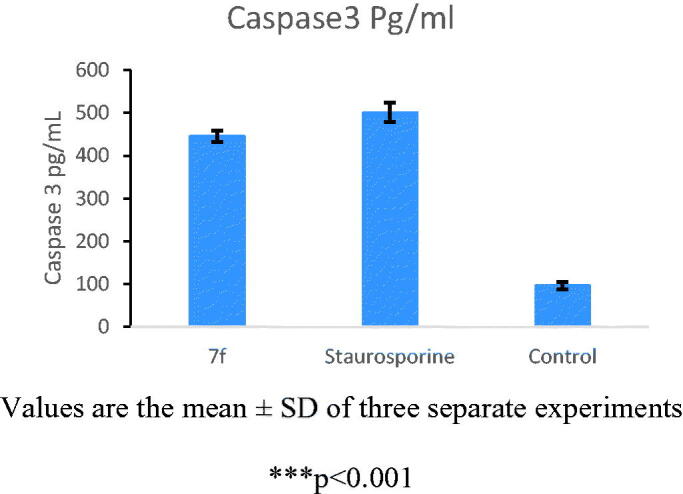
Caspase 3 levels in K562 after treatment with compound **7f**.

PI3K/AKT inhibitors are known to potentiate the intrinsic apoptotic pathway through increasing the levels of active caspase 3^38^. To further investigate the effect of the novel studied compound on caspase 3 level, K562 cells were treated with **7f** at its previously reported IC_50_ value_,_ where it showed significant increase in active caspase 3 level by approximately 5-fold (*p* < .001) as compared to the control cells. The reported result of **7f** compound was nearly the same as that of the reference compound staurosporine (protein kinase inhibitor) as shown in Figure 7.

#### 3.2.5. Western blot assay for the down-stream proteins

AKT regulates cell proliferation by stabilising cyclin D1 through GSK3β inactivation. Activated AKT phosphorylates serine 9 of GSK3β to inactivate its kinase activity on threonine 286 of cyclin D1, which then blocks the nuclear export and the cytoplasmic proteasomal degradation of cyclin D1^44^^,^^45^. AKT can also phosphorylate and activate IκB kinase (IKK), a kinase that induces degradation of the NF-κB inhibitor, IκB39. Degradation of IκB releases NF-κB from the cytoplasm, allowing nuclear translocation, and activation of the oncogenic genes^46^^,^^47^.

**Figure 8.**
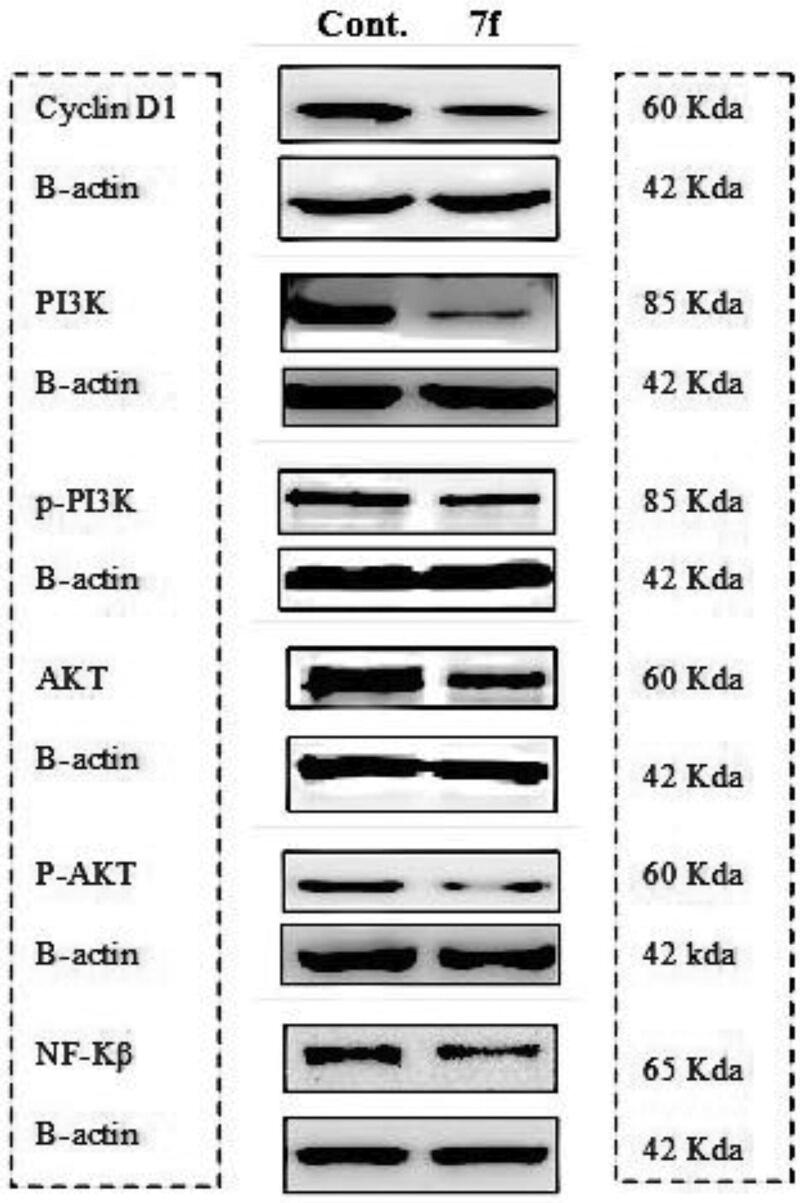
Western blot analysis of cyclin D1, Pi3k, p-Pi3k, AKT, p-AKT, and NFΚβ in leukaemia k562 cell line.

Western blot assay was conducted adopting the reported procedures^39^ to investigate the PI3K/AKT dual inhibitory activity of the most active compound **7f** on the protein expression levels of the down-stream biomarkers PI3K, AKT, and their phosphorylated forms in addition to Cyclin D1 and NF-κB in K256 cell line. The given results depicted in Table 6 and Figure 8 show a marked reduction in the expression of the investigated proteins indicating that **7f** effectively inhibits cell growth and proliferation through attenuating PI3K/AKT axis activity.

#### 3.2.6. *In-vivo* acute toxicity study

The toxicological profile of the most promising synthesised anticancer compound **7f** was evaluated in female rats using the reported method in OECD guidelines^40^. Data from this study showed that the median lethal dose (LD_50_) is greater than the test dose (2000 mg/kg) which proved that our new compound **7f** is non-toxic and is well tolerated by experimental animals.

### 3.3. *In-silico* studies

#### 3.3.1. Molecular docking

After obtaining the enzyme inhibition results on both AKT-1 and PI3K-γ, molecular simulations (docking and ADME calculations) were carried out to study the inhibitory binding of the most active compound **7f** using Discovery Studio version 4.1 software. The docking scores and the binding modes were comparable to the co-crystalised ligands.

##### 3.3.1.1. AKT-docking (ATP active site)

AKT-1 or PKBα architecture is characterised by three main domains: The *N*-terminal pleckstrin homology (PH) domain, a central serine/threonine catalytic domain (kinase domain [KD]) where the active site of ATP binding is located, and a short regulatory region called the hydrophobic motif^12^. AKT inhibitors are either ATP-competitive or Allosteric inhibitors acting where an allosteric site is created at the interface of PH and KD domains interactions that stabilise the autoinhibited enzyme. Thus, to investigate the inhibitory mechanism of the newly synthesised compound **7f** on AKT-1, the binding patterns were simulated in the ATP-binding site and the allosteric site.

For ATP competitive binding inhibition, the pdb code 4ekl^21^^,^^41^^,^^48^ was used co-crystalised with the reference compound Ipatasertib which has the typical pharmacophore of AKT inhibitors: 1) it has a heteroaromatic moiety that form hydrogen bond in the hinge region with **Ala230** and **Glu228**. 2) Aa linker/spacer connected to a basic amine interacting with **Glu234** and **Glu278** in the acidic rich pocket, and 3) a substituted aromatic ring that can fit into a hydrophobic groove formed by **Gly159**, **Lys158**, **Lys179**, and **Leu181** under the P-loop.

**Figure 9.**
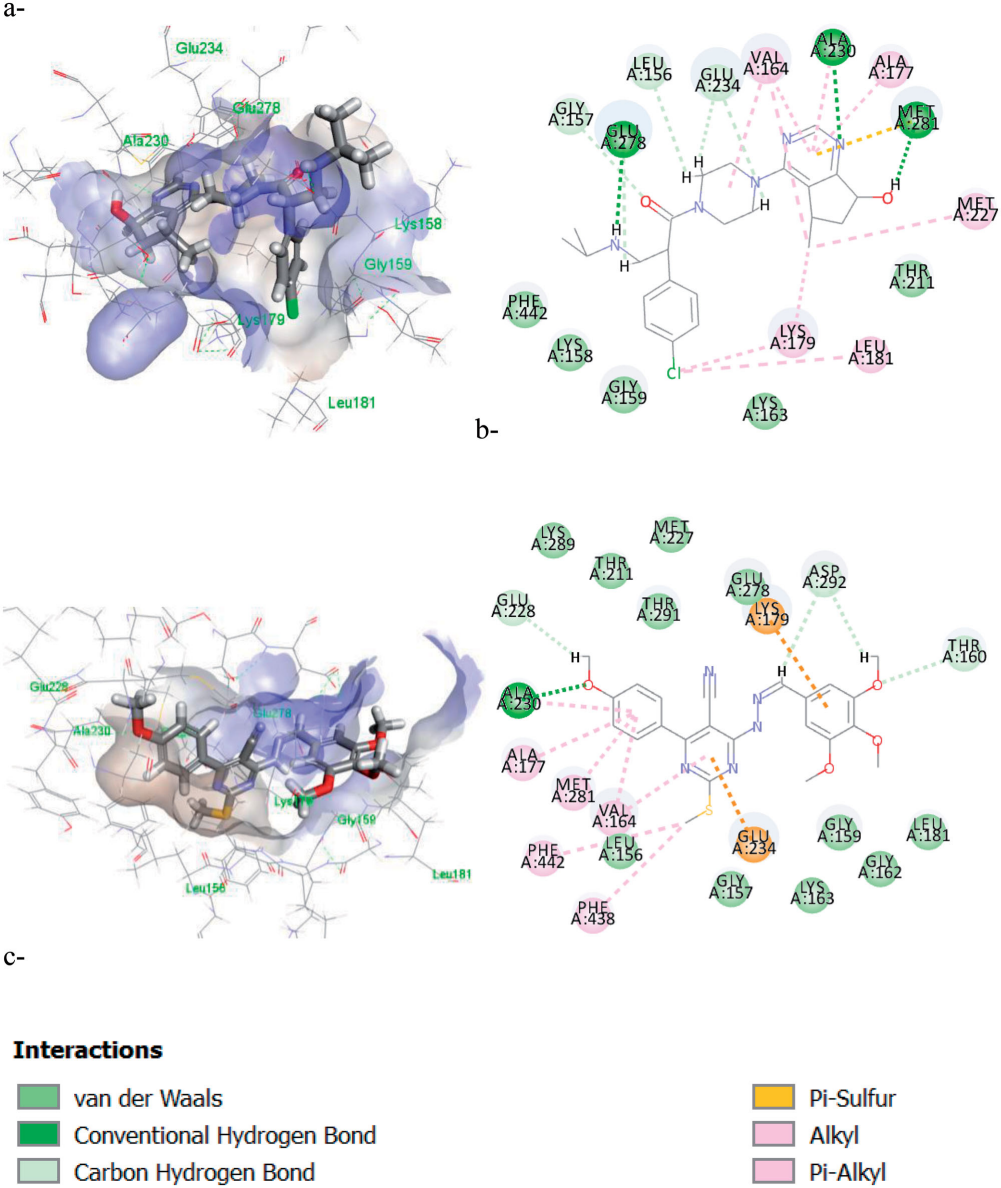
The 2D/3D interaction diagrams of: a) the reference compound (**GDC-0068**); b) **7f** binding; c) the types of binding interactions.

The 2D and 3D interaction diagrams in Figure 9 revealed similar orientation to the reference compound inside the ATP-active site where the aromatic 4-methoxyphenyl was inserted inside the hinge region (**Ala230**), the trimethoxyphenyl moiety interacts with **Lys178** in the hydrophobic pocket, and the pyrimidine interacts with **Glu234** in the acidic region. The docking scores are shown in Table 7.

**Table 7.** CDOCKER scores of the most active compound **7f** in Kcal/mol in AKT-1 (ATP-binding site).

Compound	CDOCKER score (Kcal/mol) in AKT-1 (ATP-pocket)
**GDC-0068**	−26.76/−57.76
**7f**	−14.15/−54.99

##### 3.3.1.2. AKT-docking (allosteric binding site)

Furthermore, the most active compound **7f** was docked into the allosteric binding site (pdb code:**4ejn**)^42^ of AKT-1 to study different inhibition modes. The allosteric non-ATP dependent pocket is formed by the interaction of the PH domain and the KD in AKT-1 leading to an auto-inhibited conformation. In this autoinhibited conformation, the ATP binding site is occluded by nonpolar clusters (**Ile84**) forming a hydrophobic lock that prevents ATP and ATP-competitive inhibitors from binding. The main interactions in this binding site are a) The core nucleus imidazopyridine is stacked hydrophobically between **Trp80** embedded in the PH domain, **Val270**, and **Leu264**; b) hydrogen bond interaction with N-pyridine ring and **Asp292**; c) hydrophobic interactions with **Tyr272**; d) the amide linker helps direct the terminal aromatic ring towards the hydrophobic lock.

**Figure 10.**
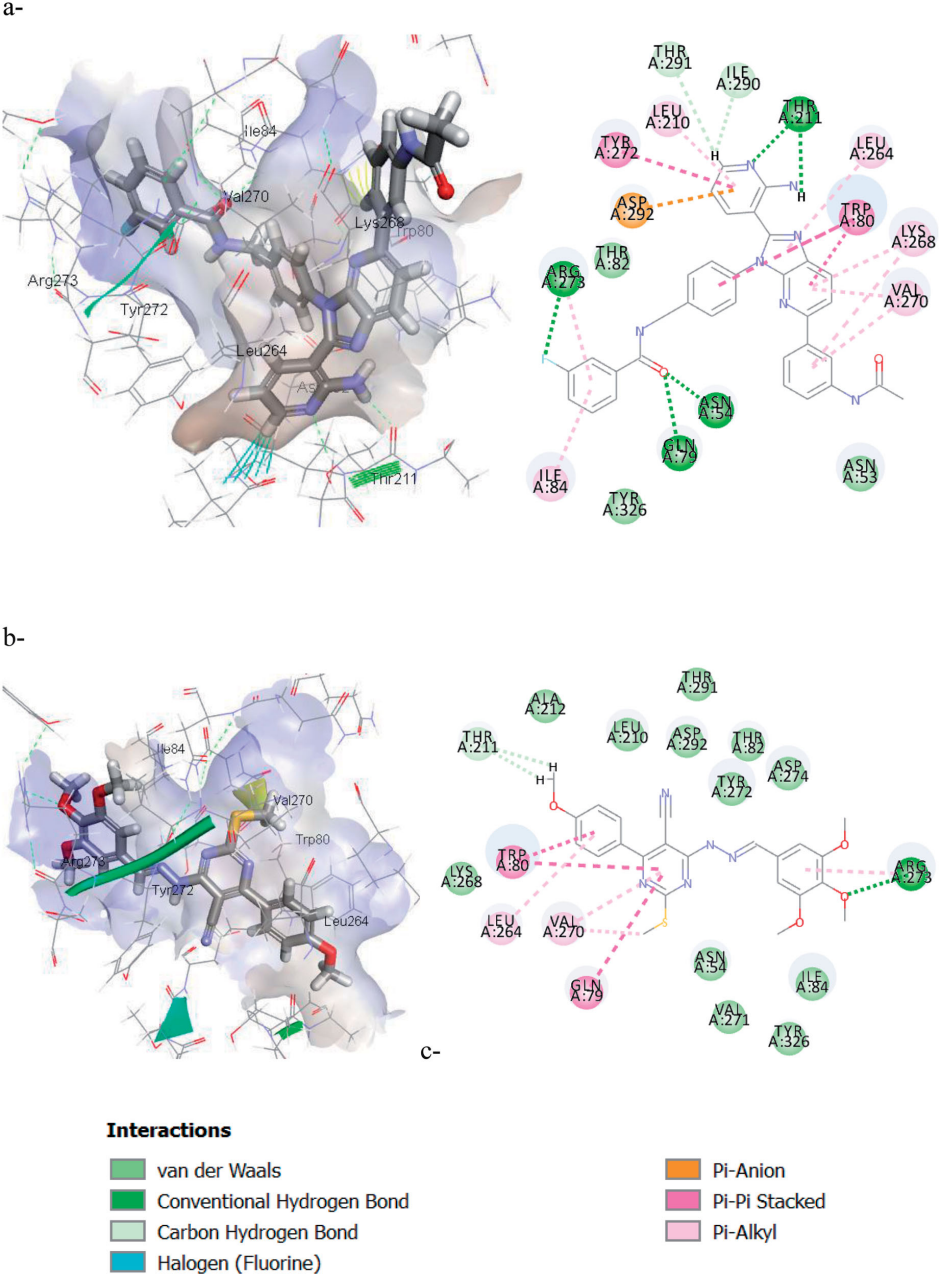
The 2D/3D interaction diagrams on allosteric site of AKT-1 of: a) the reference compound (**OR4**); b) **7f** binding.

Through studying the binding pattern of the most active compound **7f** within the AKT-1 allosteric site represented in Figure 10 and the scores in Table 8, it was found that it has a similar binding mode like the reference compound (OR4). The core pyrimidine is stacked hydrophobically between the key amino acids **Val270**, **Leu264**, and **Trp80**. Also, the hydrazono linker at C-6 helps orienting the terminal phenyl ring towards the hydrophobic lock containing **ILe84** similar to the amide linker of the reference ligand, and the trimethoxy phenyl interacts with **Arg273** similar to the *m*-fluorophenyl of the reference compound. This suggests that its mechanism of inhibiting AKT-1 isoform might be through non-competitive allosteric binding.

**Table 8.** CDOCKER scores of the most active compound (**7f**) in Kcal/mol in AKT-1 allosteric pocket.

Compound	CDOCKER score (Kcal/mol) on AKT-1 (allosteric pocket)
**Co-crystallised Ref [OR4]**	−27.95/−71.75
**7f**	−27.36/−59.43

##### 3.3.1.3. PI3K-γ docking

**Figure 11.**
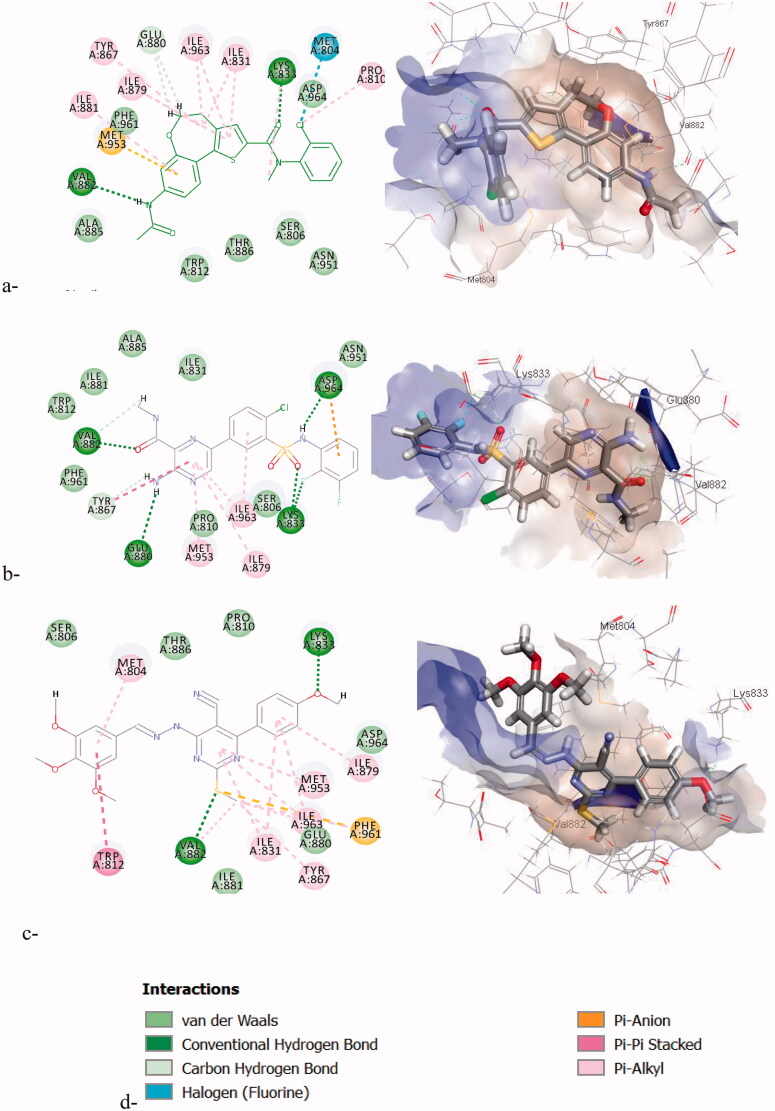
The 2D/3D interaction diagrams of: a) the co-crystalised ligand; b) the reference compound (**OR4**); c) **7f** binding in PI3Kγ.

The docking simulation of compound **7f** inside the PI3K-γ ATP-binding site (pdb code: 3r7r)^49^ revealed that the binding patterns are similar to the reference compounds with the key amino acids **Val882** in the hinge region and **Lys833** in the P-loop as illustrated in Figure 11. Also, the docking scores are comparable to the co-crystalised ligand and reference compound **O92** as presented in Table 9. These simulation studies imply that the newly synthesised compound inhibits PI3K***-****γ* through competing with ATP and AKT-1 through acting on its allosteric binding site.

**Table 9.** CDOCKER scores of the most active compound **7f** in PI3K-γ.

	CDOCKER score (Kcal/mol)
Compound	PI3K-γ
**Co-crystallised Ref (1.^8^ nM)**	−18.32/−49.64
**O92 (18 nM)**	−15.12/−47.42
**7f**	−15.63/−50.03

#### 3.3.2. Drug-likeness and molecular properties calculations

Since the biological activity of a given drug is a function of its physicochemical properties as they affect its ability to reach its biological target in reasonable concentration, it was important to determine these ADMET parameters for the newly discovered pyrimidine derivative **7f** using Discovery Studio version 3.0 suite. The presented data in Table 10 demonstrated that **7f** obeys Lipinski’s rule of five which gives an indication about its drug-likeness. It has a Log *P* of 5 that may reflect on the low solubility, absorption levels and enhances the blood–brain barrier penetration. Additionally, it has plasma proteins binding ability due to its lipophilicity which will affect its duration of action, half-life, and elimination. Also, it was estimated that it is not a substrate for CYP2D6 therefore there will probably not be drug–drug interactions. Finally, the AMES and hepatotoxicity prediction suggested its safety^50^^,^^51^.

**Table 10.** Physicochemical properties of compound **7f** compared to Ipatasertib.

Properties	Compound (**7f**)	Reference (Ipatasertib)**^,^***
Molecular weight	465	458
HBD	1	2
HBA	10	6
Rotatable bonds	9	6
LogP98^a^	5.03	3.1
PSA^b^	105.31	80.15
Solubility level^c^	2	2
Absorption level^d^	2	0
CYP2D6	False	False
Plasma protein binding	true	False
BBB level	4	2
AMES prediction	Non-mutagen	Non-mutagen
Hepatotoxicity	False	False

**The extra modifications: 10 or less rotatable bonds and polar surface area (PSA) equal to or less than 140 Å^2^.

***^a^Lipophilicity descriptor; ^b^polar surface area; ^c^solubility level (0 = extremely low, 1 = very low but soluble, 2 = low, 3 = good, and 4 = optimal); ^d^absorption level (0 = good, 1 = moderate, 2 = low, and 3 = very low).

## 4. Conclusions

Innovative dual-acting PI3K/AKT inhibitors bearing 4-(4-methoxyphenyl)pyrimidine were synthesised and evaluated for their antiproliferative activity. All target compounds were screened for their cytotoxicity against breast cancer (MCF-7) and leukaemia (K562) cell lines. They demonstrated promising activities especially compounds **4d** and **7f** that exhibited excellent activities on both cell lines. The derivative **7f** showed a strong effect in inhibiting PI3K and AKT enzymes as well as decreasing their protein expression. Furthermore, compound **7f** caused cell cycle arrest at S phase leading to induction of apoptosis in leukaemia (k562) cells through caspase 3 activation by 5-fold compared to the control. It also showed a multi-protein targeting through preventing the downstream expression of Cyclin D1and NF-Κβ, in addition to the non-toxic profile through the *in-vivo* toxicity study and ADMET prediction. Simulation studies proved that the design strategy adopted was efficient to develop dual enzyme inhibitor where the PI3K-γ inhibition *via* ATP-competitive inhibition and AKT-1 through acting on the allosteric binding site. In conclusion, the newly synthesised pyrimidine compounds are effective anticancer leads through inhibition of PI3K/AKT axis and caspase 3 dependent apoptosis induction with a potential for advanced optimisation in future work.

### 4.1. Statistical analysis

Data are reported as means ± SD. Statistical analysis was performed using the GraphPad Prism version (GraphPad Software, La Jolla, CA). A *p* value less than .05 was considered statistically significant.

### 4.2. Animal use

All experimental operations performed on rats were approved by the Animal Experiment Ethics Committee of Heliopolis University (Approval number HU.REC.A0.23-2021).
